# Development of a Versatile Nanostructured Lipid Carrier (NLC) Using Design of Experiments (DoE)—Part II: Incorporation and Stability of Butamben with Different Surfactants

**DOI:** 10.3390/pharmaceutics16070863

**Published:** 2024-06-27

**Authors:** Ananda P. Matarazzo, Carlos A. Rios, Gabriela Gerônimo, Roberta Ondei, Eneida de Paula, Márcia C. Breitkreitz

**Affiliations:** 1 Faculty of Pharmaceutical Sciences, University of Campinas (UNICAMP), Campinas 13083-871, SP, Brazil; a237288@dac.unicamp.br; 2 Institute of Chemistry, University of Campinas (UNICAMP), Campinas 13083-862, SP, Brazil; c228209@dac.unicamp.br; 3 Department of Biochemistry and Tissue Biology, Institute of Biology, University of Campinas (Unicamp), Campinas 13083-862, SP, Brazil; g226533@dac.unicamp.br (G.G.); depaula@unicamp.br (E.d.P.); 4 Croda Brazil, R. Croda, 580—Distrito Industrial, Campinas 13054-710, SP, Brazil; roberta.ondei@croda.com

**Keywords:** nanostructured lipidic carriers, design of experiment, butamben

## Abstract

Nanostructured lipid carriers (NLCs) are typically composed of liquid lipids, solid lipids, and surfactants, enabling the encapsulation of lipophilic drugs. Butamben is a Class II anesthetic drug, according to the Biopharmaceutical Classification System (BCS); it has a log P of 2.87 and is considered a ‘brick dust’ (poorly water-soluble and poorly lipid-soluble) drug. This characteristic poses a challenge for the development of NLCs, as they are not soluble in the liquid lipid present in the NLC core. In a previous study, we developed an NLC core consisting of a solid lipid (Crodamol^TM^ CP), a lipophilic liquid with medium polarity (SR^TM^ Lauryl lactate), and a hydrophilic excipient (SR^TM^ DMI) that allowed the solubilization of ‘brick dust’ types of drugs, including butamben. In this study, starting from the NLC core formulation previously developed we carried out an optimization of the surfactant system and evaluated their performance in aqueous medium. Three different surfactants (Crodasol^TM^ HS HP, Synperonic^TM^ PE/F68, and Croduret^TM^ 40) were studied and, for each of them, a 2^3^ factorial design was stablished, with total lipids, % surfactant, and sonication time (min) as the input variables and particle size (nm), polydispersity index (PDI), and zeta potential (mV) as the response variables. Stable NLCs were obtained using Crodasol^TM^ HS HP and Synperonic^TM^ PE/F68 as surfactants. Through a comparison between NLCs developed with and without SR^TM^ DMI, it was observed that besides helping the solubilization of butamben in the NLC core, this excipient helped in stabilizing the system and decreasing particle size. NLCs containing Crodasol^TM^ HS HP and Synperonic^TM^ PE/F68 presented particle size values in the nanometric scale, PDI values lower than 0.3, and zeta potentials above |10|mV. Concerning NLCs’ stability, SBTB-NLC with Synperonic^TM^ PE/F68 and butamben demonstrated stability over a 3-month period in aqueous medium. The remaining NLCs showed phase separation or precipitation during the 3-month analysis. Nevertheless, these formulations could be freeze-dried after preparation, which would avoid precipitation in an aqueous medium.

## 1. Introduction

The use of low water solubility drugs in the development of pharmaceutical formulations has motivated research related to the development of new delivery and release systems, which must be capable of increasing their bioavailability and absorption, regardless of the route of administration [1,2,3]. Drugs exhibiting poor solubility in water are generally classified according to the Biopharmaceutical Classification System (SCB) as belonging to Classes II and IV. This classification considers two key physicochemical parameters: aqueous solubility and intestinal permeability [4,5].

Drugs belonging to Class II present low aqueous solubility and high permeability in cell membranes; in this case, in addition to solubility, their dissolution rate will affect their absorption by the gastrointestinal tract, as at this stage of the absorption process the medicine must be solubilized to be absorbed [6,7]. This is the case for butamben, which, besides being classified as Class II, also exhibits ’brick dust’ behavior, indicating poor solubility in water and limited solubility in lipids [5,7,8,9]. One of the ways to improve the absorption and bioavailability of these types of drugs is through incorporation into nanostructured lipid carriers (NLCs), which increase drug absorption. NLCs reduce formulation particle size, leading to an increase in surface area, which results in a greater exposure of lipid fractions in epithelial membranes. This promotes bioadhesion to the gastrointestinal walls, prolonging their residence time in the gastrointestinal tract and thus increasing their absorption [5,7,8,9].

Nanostructured lipid carriers consist of a solid lipid matrix (biodegradable and biocompatible), containing a mixture of solid and liquid lipids dispersed in an aqueous phase containing surfactants. [10,11]. NLCs are considered second-generation carriers, as they present a variation in their internal structure when compared to first-generation carriers (solid lipid nanoparticles, SLNs), attributed to the introduction of a liquid lipid. This structural variation disrupts the order of solid lipid crystals, resulting in an increase in drug accommodation spaces and inhibiting drug crystallization. Furthermore, the presence of solid lipids also contributes to inhibiting the drug crystallization process. Due to this, NLCs achieve greater drug incorporation with higher encapsulation efficiency and enhanced stability of the lipid matrix [10,12].

The use of experimental design and optimization (DOE) methods allows a reduction in the number of experiments required in order to determine the optimal amount of excipients and, therefore, achieve an adequate and stable formulation in the face of different quality attributes [13,14]. The DOE is a systematic tool in which it is possible to determine the relationships between the input (x) and output (Y) variables, thus providing a better understanding of the process and the optimization of this process. Through DOE, it is possible to optimize the developed formulations, thus generating results that demonstrate the best process conditions and proportion of excipients to be used to obtain greater quality and effectiveness of the product [13].

Several works have been carried out associating the development of lipid formulations such as nanostructured lipid carriers with strategies that employ the use of DOE. In these articles, the DOE technique is used to optimize the formulation by investigating the effect of changes in the proportion of the solid lipid, liquid lipid, surfactant, and co-surfactant on the important characteristics of the NLC formed, as well as the possible influence of the manufacturing process and drug concentration on the quality of the final product. The in vitro responses used were particle size, PDI, zeta potential and encapsulation efficiency [15,16,17,18,19,20,21,22].

The present paper describes the second part of a study [1] in which Raman microscopy and DOE were used to evaluate the miscibility between excipients candidates for a medium polarity lipidic core. Thus, it was determined that the ideal mixture would be composed of Crodamol CP^TM^ (40% *w*/*w*), Lauryl Lactate^TM^ (25% *w*/*w*), and DMI^TM^ (25% *w*/*w*), as they presented adequate miscibility with one another and also a good solubilization capacity for butamben (BTB), a brick dust type of drug.

This study evaluated three different types of surfactants for NLC formation in aqueous medium, Crodasol^TM^ HS HP, Synperonic^TM^ PE/F68, and Croduret^TM^ 40, and looked into more details of the use of Super Refined^TM^ DMI (SR^TM^ DMI) within the lipid core.

## 2. Materials and Methods

### 2.1. Excipients

The excipients used were Crodamol™ CP pharma (Code: ES68635, Batch: 00017585465, INCI name: Cetyl Palmitate, Campinas, São Paulo, Brazil), Super Refined™ Lauryl Lactate (Code: SR41856, Batch: 0001842276, INCI name: Lauryl Lactate, Mill Hall, PA, USA), Super Refined™ DMI (Code: SR40492, Batch: 0001796734, INCI name: Dimethyl Isosorbide, Mill Hall, PA, USA), Croduret™ 40 (Code: ET02113, Batch: 0001833931, INCI name: PEG-40 hydrogenated castor oil, Rawcliffe Bridge, East Riding of Yorkshire, UK), Crodasol™ HS HP (Code: ET40486, Batch: 0001773939, INCI name: Macrogol 15 Hydroxystearate, Mill Hall, PA, USA), and Synperonic^TM^ PE/F68 (Code: ETK1229, Batch: 2101AS3909, INCI name: Poloxamer 188, Campinas, São Paulo, Brazil). All excipients were donated by Croda do Brazil (Campinas, SP, Brazil). All other chemicals and solvents were of analytical grade.

### 2.2. Preparation of Butamben (BTB)-Loaded NLCs

The nanostructured lipid carrier formulations were prepared using the emulsification-ultrasonication method [23]. The technique involves adding the aqueous phase to the oil phase under agitation, followed by sonication. To prepare the NLC, the lipid phase, composed of Crodamol^TM^ CP, Super Refined^TM^ Lauryl Lactate, and Super Refined™ DMI, along with butamben (5% *w*/*w*), was weighed and heated in a water bath to 65 °C. Simultaneously, the aqueous phase, consisting of one of the selected surfactants and 10 mL of MilliQ water, was heated to the same temperature as the lipid phase. The aqueous phase was then added to the lipid phase to form the pre-emulsion, with stirring at 11,000 rpm for 3 min using an Ultra-Turrax (T18, IKA WerkeStaufen, Staufen, Germany). The pre-emulsion formed underwent tip sonication, involving ultrasonic agitation with a titanium microtip (Vibracell equipment Sonics & Mat. Inc., Danbury, CT, USA), at an amplitude of 70% and 20 KHz nominal frequency, for 5 or 10 min with alternating cycles of 30 s (on–off). The same procedure was followed for the preparation of drug-free NLCs.

### 2.3. Experimental Design and Optimization of BTB-Loaded NLCs

For NLC formulations, the following variables were studied through a 2^3^ full factorial design with triplicates at the center point: % total lipids, % surfactant, and a process variable associated with the emulsification-ultrasonication method, the sonication time (min) (Table 1). Butamben concentration was fixed at 5%. The objective of this step was to identify the most promising proportions of the selected excipients and evaluate these formulations based on critical quality attributes: particle size, polydispersity, and zeta potential.

The factorial design shown in Table 1 was carried out for the three selected surfactants (Crodasol^TM^ HS HP, Synperonic^TM^ PE/F68, and Croduret^TM^ 40). Statistical analysis of the results was utilized to examine the influence of each excipient on each response and desirability functions were used to achieve the overall optimization using the software Design Expert version 13 (Stat-Ease Inc., Minneapolis, MN, USA).

The optimized NLCs determined by the DOE were prepared with and without butamben to carry out freeze-drying for further Differential Scanning Calorimetry (DSC) and Infrared Spectroscopy (FT-IR) analyses. Furthermore, formulations were prepared with and without the inclusion of SR^TM^ DMI to investigate its influence on the NLC system.

### 2.4. Characterization of Butamben-Loaded NLCs

#### 2.4.1. Particle Size, Polydispersity Index (PDI), and Zeta Potential (ZP)

Particle size and zeta potential are important parameters for the physical stability of dispersed systems. The average particle size, polydispersity index (PDI), and zeta potential were determined by dynamic light scattering (DLS) using the Zetasizer Nano ZS 90 analyzer (Malvern Instruments, Worcestershire, UK). Determinations were carried out at 25 °C. Zeta potential was determined by employing the laser Doppler electrophoresis method. An aliquot of the freshly prepared dispersion was dispersed in deionized water and diluted 1000× for particle size and zeta potential analyses.

#### 2.4.2. Encapsulation Efficiency

Drug encapsulation efficiency (EE) corresponds to the percentage of drug encapsulated and absorbed into the particles. EE was indirectly determined by the ultrafiltration-centrifugation method, using cellulose filters (Amicon Ultra-Millipore 10 KDa ultrafiltration devices, Merk Millipore Ltd., Carrigtwohill, Co Cork, Ireland). An amount of 400 µL of prepared SBTB-NLC dispersion was added to the upper part of the device and centrifuged at 4100× *g* for 20 min. Ultra-High-Performance Liquid Chromatography (UHPLC) was used to determine the amount of free drug in the filtrate, and the total drug in the dispersion was determined by dissolving an aliquot of the dispersion in acetonitrile, followed by UPLC analysis. The encapsulation efficiency was determined using the following Equation (1):

*EE (%) = (total drug − free drug)/(total drug)* × 100 (1)

#### 2.4.3. Nanoparticle Tracking Analysis

Nanoparticle tracking analysis (NTA) was conducted to determine the size, distribution, and concentration of nanoparticles in the developed formulations, providing a real-time concentration of nanoparticles (particles/mL) [24,25]. Measurements were performed using the NS300 equipment (NanoSight, Amesbury, UK), which was equipped with a 532 nm laser. The samples were diluted in deionized water (50,000×) and then transferred to a sample holder with a syringe. Data are expressed as the mean ± standard deviation, and measurements were performed in triplicate. Values for Span were calculated using Equation (2):

(2) $$Span=\frac{D90-D10}{D50}$$

where D90, D50, and D10 are the average diameter of 90%, 50%, and 10% of the particle population, respectively.

#### 2.4.4. Differential Scanning Calorimetry (DSC)

Differential scanning calorimetry measurements were performed using the DSC Q100 equipment (TA Instruments, New Castle, DE, USA). For analysis, the samples were placed in hermetically sealed aluminum crucibles. The test was conducted under a dynamic argon atmosphere (50 mL/min) with a heating rate of 10 °C/min and a heating range of 10 to 220 °C. The following samples were analyzed: lyophilized NLCs (SBTB-NLC (NLC with Synperonic^TM^ PE/F68 and BTB), CBTB-NLC (NLC with Crodasol^TM^ HS HP and BTB), S-NLC (NLC with Synperonic^TM^ PE/F68 without BTB), and C-NLC (NLC with Crodasol^TM^ HS HP without BTB)), the drug (butamben), the solid lipid (Crodamol^TM^ CP), and the surfactants (Crodasol^TM^ HS HP and Synperonic^TM^ PE/F68).

#### 2.4.5. Fourier Transform Infrared-Attenuated Total Reflectance

FTIR spectra were obtained for the lyophilized formulations (SBTB-NLC, CBTB-NLC, S-NLC, and C-NLC), the drug (butamben), the solid lipid (Crodamol^TM^ CP), and the surfactants (Crodasol^TM^ HS HP and Synperonic^TM^ PE/F68). The equipment used was an Agilent Cary 630 Fourier transform infrared spectrophotometer (Agilent, Victoria, Australia), with attenuated total reflectance cell (ATR-FTIR), 128 scans per analysis, and a resolution of 2.0 cm^−1^, in the range of 4000 to 400 cm^−1^.

### 2.5. Three-Month Stability Study of the Optimized NLCs

Based on the definition of the optimized NLCs, an accelerated stability study of pilot formulations was conducted for a period of up to 3 months, after which the formulations were re-evaluated for particle size, PDI, and zeta potential. The stability test was carried out with NLCs containing BTB, with SR^TM^ DMI, and without SR^TM^ DMI.

## 3. Results and Discussion

### 3.1. Factorial Designs

As mentioned in the methodology, the selection of excipients for the NLC core and their proportions were determined in a previous study through Raman mapping and DOE [1]. From this previous study, it was established that Crodamol^TM^ CP would be used as a solid lipid, SR^TM^ DMI, and SR^TM^ Lauryl Lactate as a liquid lipid (SR^TM^ DMI is indeed a solvent; however, since it goes within with lauryl lactate, it will be denoted here ‘liquid lipid’ as well), and several drugs were evaluated, including butamben. The proportions between Crodamol^TM^ CP and Super Refined^TM^ Lauryl Lactate were set at 1:1.6 (*w*/*w*), and the ratio of SR^TM^ DMI to Super Refined^TM^ Lauryl Lactate was 1:1 (*w*/*w*).

The 2^3^ factorial design aimed at identifying the optimal percentage of total lipids, the most effective surfactant and its percentage (critical material attributes—CMAs), and the optimal sonication time (critical process attribute—CPP) that would allow the achievement of suitable particle size, PDI, and zeta potential—considered critical quality attributes (CQAs). Table 2 outlines the selected independent and dependent variables along with the specified objectives for particle size, PDI, and zeta potential.

The selected surfactants for the study were Crodasol^TM^ HS HP, Synperonic^TM^ PE/F68, and Croduret^TM^ 40. Through the 2^3^ design for each surfactant, 11 different experimental combinations were obtained for formulations and subsequent analysis (Table 3).

#### 3.1.1. Interpretation of Responses Obtained for the DOE Using Crodasol^TM^ HS HP as Surfactant

**Figure 1:**
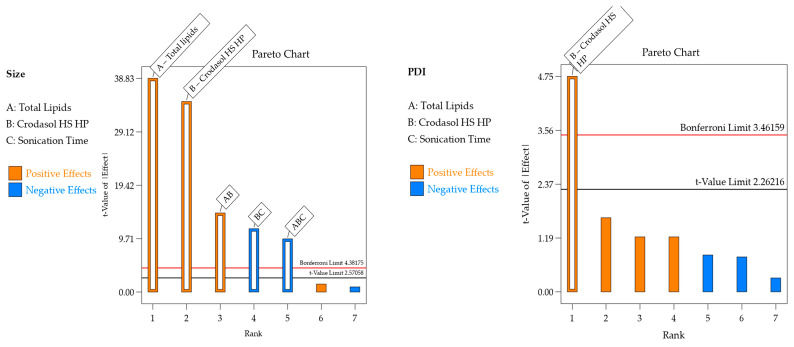
Pareto chart with significant effects and their interactions in each response for the formulation containing Crodasol^TM^ HS HP as surfactant.

**Figure 2:**
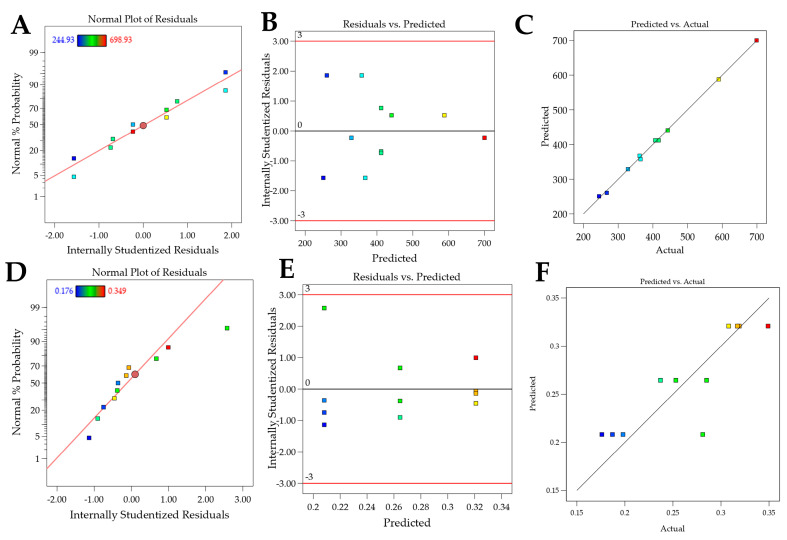
Graphs of residual analyses for models containing Crodasol^TM^ HS HP as a surfactant. (**A**–**C**): residual analysis for particle Size; (**D**–**F**): residual analysis for the PDI response.

The responses obtained for the design with Crodasol^TM^ HS HP as a surfactant are presented in Table 4. The regression was significant for particle size and PDI and not for zeta potential, i.e., the deliberate experimental variations did not affect this response. Linear models were well adjusted for both responses. The effect of excipients on the responses of interest is detailed in Table 4 and Figure 1, and the residue analysis of linear models is depicted in Figure 2.

As can be seen in Table 4, the formulation particle size ranged from 244.93 nm to 698.93 nm, the PDI from 0.176 to 0.349, and the zeta potential from 25.66 to 29.26.

For the particle size response, the R^2^ was 0.9987. Residual analysis graphs (Figure 2A,B) indicated that the residuals follow a normal distribution, are homoscedastic and independent. Additionally, Figure 2C shows an excellent agreement between the predicted and actual values, thus indicating the model’s good predictive capability. As described in Table 5, the particle size response exhibited two significant main effects and three significant interactions. Analyzing the significant effects, Crodasol^TM^ HS HP, % surfactant (X_2_), and the total lipid (X_1_) variables showed positive effects. The positive effect of the surfactant was unexpected since, typically, in such formulations, the surfactant effect is negative, i.e., as the concentration increases, the size decreases. A possible explanation is that higher concentrations of Crodasol^TM^ HS HP result in a predominance of hydrophobic interactions due to non-polar groups, such as the long alkyl chains of the hydroxyl stearate portion of the surfactant, and the interactions between the lipids and the surfactant, causing an increase in size [26]. This outcome was also observed by Almousallam and collaborators, who used Kolliphor^®^ P 188 (Sigma Aldrich, Gillingham, Dorset, UK) as a surfactant [27]. The positive effect of the total lipids was attributed to the increase in the lipid concentration causing lipid particle coalescence, and therefore an increase in particle size [26].

For the PDI response, the R^2^ was 0.7348. The residual analysis graphs (Figure 2D–F) show that the model residuals follow a normal distribution and are homoscedastic and independent. As indicated in Table 5, the PDI response showed only surfactant (X_2_) as having a significant effect. The increase in the concentration of Crodasol^TM^ HS HP is responsible for the increase in the PDI of the formulations; this increase may be because the increase in surfactant causes particle aggregation, resulting from the surfactant binding on the surface of the nanoparticle [28].

**Figure 3:**
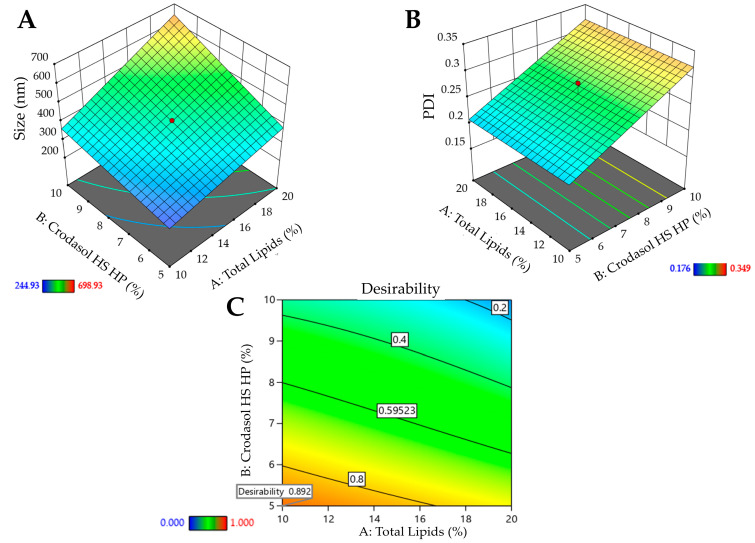
Surface graphs for the responses of the formulation containing Crodasol^TM^ HS HP as surfactant: (**A**)—particle size. (**B**)—PDI; (**C**)—desirability graph.

Figure 3A shows the 3D surface and it becomes evident that particle size is influenced by the interaction between variables X_1_ and X_2_. Higher levels of the variables X_1_ and X_2,_ result in larger particle sizes, whereas lower levels are associated with smaller particle sizes. Consequently, to achieve smaller particle sizes (indicated by the blue region in the graph), it would be ideal to work with a lower % of surfactant (5%), a lower % of total lipids (10%), and a shorter sonication time (5 min). The shorter sonication time is due to the interaction between this variable and variable X_2_. Figure 3B shows the surface graph, which clearly indicates that the PDI is influenced solely by variable X_2_. Therefore, to achieve lower PDIs (indicated by the blue region in the graph), it is advisable to work with a lower % of surfactant (5%).

After processing the data and generating the mathematical model for each property of interest (particle size, PDI, and zeta potential), it was possible to define the optimized formulation. Desirability was used with the following criteria for the responses: minimize particle size and PDI and maximize zeta potential. The desirability graph indicates the proportion that best meets the desired criteria (the closer to one the better). For the formulation containing Crodasol^TM^ HS HP as a surfactant, the desirability was 0.892 (Figure 3C). Therefore, for the Crodasol^TM^ HS HP surfactant, the optimized formulation was defined with 10% total lipids, 5% surfactant, and 7.5 min of sonication time. The formulation was prepared again and monitored in the stability study, with subsequent characterization.

#### 3.1.2. Interpretation of Responses Obtained for the DOE Using Synperonic^TM^ PE/F68 as Surfactant

**Figure 4:**
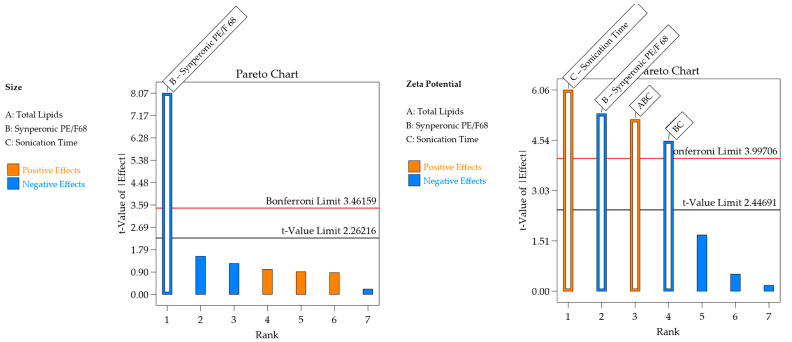
Pareto chart with significant effects and their interactions in each response for the formulation containing Synperonic^TM^ PE/F68 as surfactant.

The obtained responses for the design using Synperonic^TM^ PE/F68 as a surfactant are presented in Table 6. The effect of excipients on the responses of interest is detailed in Table 7 and Figure 4. The models were significant for particle size and zeta potential, whereas the experimental variations did not affect PDI. This indicates the robustness of the CQAs regarding this parameter. The lack of fit was nonsignificant for particle size and zeta potential.

**Figure 5:**
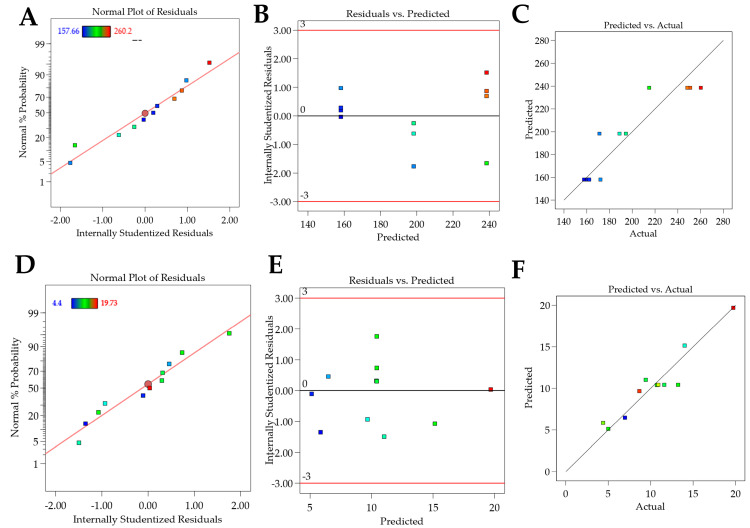
Graphs containing residue analyses for each planning response containing Synperonic^TM^ PE/F68 as surfactant. (**A**–**C**): residue analysis referring to the particle size response. (**D**–**F**): residue analysis referring to the zeta potential response.

**Figure 6:**
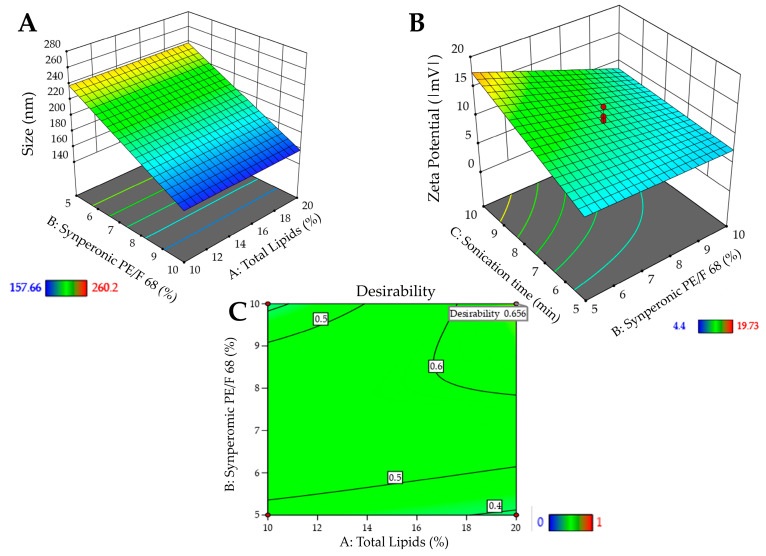
Surface graphs for the responses of the formulation containing Synperonic^TM^ PE/F68 as surfactant: (**A**)—particle size. (**B**)—zeta potential; (**C**)—desirability graph.

For the particle size, the R^2^ was 0.8465. The residual analysis graphs (Figure 5A,B) show that the model residuals follow a normal distribution, are homoscedastic and independently distributed. Additionally, Figure 5C demonstrates a good agreement between predicted and actual values, indicating the good predictive capacity of the model. The particle size of the formulations ranged from 157 nm to 260 nm and, as shown in Table 7 and Figure 4, only the surfactant Synperonic^TM^ PE/F68 (X_2_) has a significant (negative) effect. This negative effect for the surfactant is expected, as the surfactant reduces the surface tension between the lipid and aqueous phases, resulting in the formation of smaller nanoparticles and preventing the coalescence of larger droplets [15,28]. This is clear when analyzing Figure 6A, which includes the surface graphs. Therefore, to obtain smaller particle sizes (indicated by the blue region in the graph), it would be optimal to work with a higher % of surfactant (10%).

For the zeta potential response, the R^2^ was 0.9116. In the residual analysis graphs (Figure 5D,E), it is observed that the model residuals follow a normal distribution, are homoscedastic, and are independently distributed. Figure 5F shows a good agreement between predicted and actual values, thus indicating the good predictive capability of the model. As shown in Table 7 and Figure 4, the zeta potential response presented surfactant and sonication time as having significant effects. The zeta potential of the formulations varied between |4| mV and |19.7| mV.

When analyzing Figure 6B, which includes the surface graphs, it is noted that the variables that affect the zeta potential are surfactant (X_2_) and sonication time (X_3_). The variable X_2_ is associated with an increase in zeta potential when used at its lowest level and the variable X_3_ is associated with an increase in zeta potential when used at its highest level. Therefore, to obtain higher potentials, it would be optimal to work with a lower % of surfactant (5%), a lower % of total lipids (10%), and a longer sonication time (10 min).

For the formulation containing Synperonic^TM^ PE/F68 as a surfactant, the desirability was 0.656 (Figure 6C). It is worth noting that Figure 6C is shown in full green, as the particle size effect presented the desirability criterion (size smaller than 260 nm) at all tested points. Therefore, for the surfactant Synperonic^TM^ PE/F68, the optimized formulation was defined with 20% total lipids, 10% surfactant, and 10 min of sonication time. The formulation was prepared again and monitored in the stability study, with subsequent characterization.

#### 3.1.3. Interpretation of Responses Obtained for the Experimental Design Using Croduret^TM^ 40 as Surfactant

The initial objective was to carry out experimental designs with all the surfactants selected for the project. However, when using Croduret^TM^ 40 in the formulations, phase separation occurred shortly after preparation, indicating that Croduret^TM^ 40 could not form stable NLCs. This could have happened due to its structure; it is an ethoxylated surfactant of plant origin, non-ionic, with an HLB of 13, normally used as an excipient in self-emulsifying drug delivery systems (SEDDS) [29]. Therefore, studies with this surfactant were not continued.

### 3.2. Comparison between Formulations with Crodasol^TM^ HS HP, and Synperonic^TM^ PE/F68

Table 8 presents a summary of the significant effects found for each response in each design, allowing a comparison between the use of different types of surfactants. The variation of the factors was consistent (Table 2 and Table 3). Therefore, the same % total lipids, % surfactant, and sonication time were consistently utilized.

Synperonic^TM^ PE/F68 (the same chemical structure of Poloxamer 188) consists of triple copolymers formed by Polyethylene glycol-b-polypropylene glycol-polyethylene glycol (PEO-PPO-PEO)—with 16% PPO and 84% PEO—has an HLB > 24, and provides a greater number of solubilized chains both in the lipid core and in the aqueous phase, whereas Crodasol (70% of polyglycol mono and diesters of 12-hydroxystearic acid and 30% free polyethylene glycol, synonymous with Macrogol 15-Hydroxystearate), despite having a high HLB and a capability to reduce interfacial tension and also form micelles, has smaller chains, enhancing the formation of larger particles with a higher PDI and thus lower stability, as proven by the stability test.

**Figure 7:**
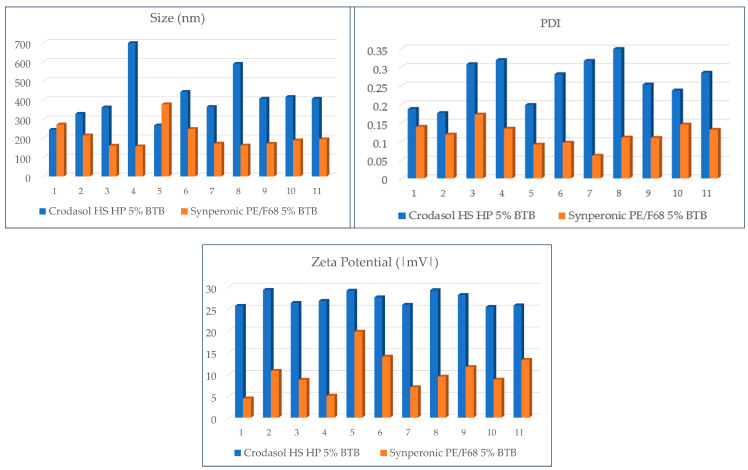
Size, PDI, and zeta plots of the DOE formulations with the two surfactants, containing BTB. Zeta Potential Values are expressed in the module.

It is observed that the difference in chemical composition between the two surfactants is also reflected in the values of particle size, PDI, and zeta potential. Figure 7 displays the size, PDI, and zeta graphs of the three surfactants used in formulations with BTB.

Analyzing Figure 7, it was observed that formulations with the Crodasol^TM^ HS HP surfactant resulted in a larger particle size (244 nm–698 nm), higher PDI values (0.176–0.319), and elevated zeta potential values (|25–29|) in comparison to formulations containing Synperonic^TM^ PE/F68 (size: 157–260 nm. PDI: 0.091–0.172. Zeta: |4.4–19| mV) as the surfactant.

Regarding the difference in particle size between Crodasol^TM^ HS HP and Synperonic^TM^ PE/F68, it can be influenced by the hydrophilic–lipophilic balance (HLB) value. Crodasol^TM^ HS HP has an HLB of 15, while Synperonic^TM^ PE/F68 has an HLB of >24. Since the solid lipid used is Crodamol^TM^ CP, characterized by high hydrophobicity and molecular weight, it contributes to the viscosity of the system. Consequently, this requires a surfactant with a higher HLB [30].

### 3.3. Characterization of Nanostructured Lipid Carriers with BTB

After determining the proportions of total lipids, liquid lipid, and sonication time for each surfactant, the optimized formulations were characterized and their stability was evaluated. Additionally, the optimized formulations were reproduced without the presence of SR^TM^ DMI to investigate the possible influence of the solvent on the nanoparticles. Table 9 presents the optimized formulations that were characterized, while Table 10 details the amount of each excipient used in the formulations without SR^TM^ DMI.

### 3.4. Particle Size Analysis, Polydispersity Index, and Zeta Potential Determination

The optimized formulations, as outlined in Table 11 and Table 12, were characterized by particle size, PDI, and zeta potential.

**Figure 8:**
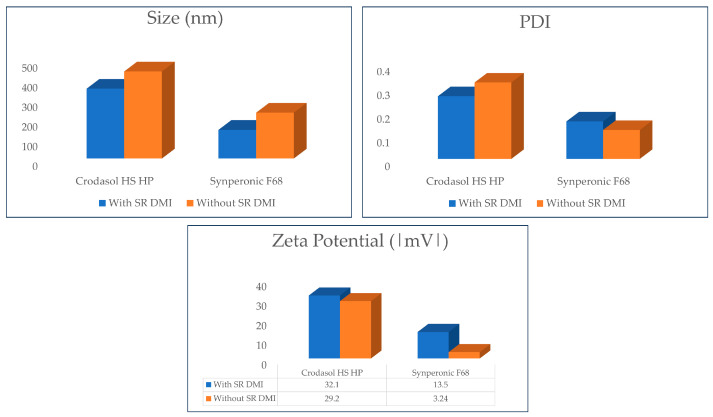
Graphical presentation of the particle size, PDI, and zeta potential results of the optimized formulations with and without SR^TM^ DMI.

Initially, the formulations were analyzed both with and without SR^TM^ DMI in the presence of BTB. These results are presented in Figure 8.

It can be observed that the removal of SR DMI^TM^ from the formulations changed particle size and zeta potential, except for the formulation containing Crodasol^TM^ HS HP as a surfactant, in which the zeta potential of the system remained unchanged. The optimized formulations without SR^TM^ DMI exhibited larger particle sizes and lower zeta potentials, generating potentially less stable particles. NLCs with SR^TM^ DMI having smaller particle sizes may suggest that SR^TM^ DMI preferred to remain in the lipid nucleus, alongside Super Refined^TM^ Lauryl Lactate, a compound of medium polarity, rather than leaving the nucleus and entering the aqueous phase of the NLC. Therefore, it can be inferred that SR^TM^ DMI helped stabilizing the system.

Studies of particle size, PDI, and zeta potential with the optimized formulations without the presence of BTB were also carried out. Results are shown in Table 12.

Table 12 shows that the size of NLC particles containing BTB is larger than that of empty NLCs, and this increase in size may indicate drug encapsulation within the particles. The PDI of NLCs with BTB (CBTB-NLC and SBTB-NLC) decreased slightly compared to S-NLC and C-NLC, remaining below 0.3, which indicates a uniform size distribution. The zeta potential of NLCs with BTB also decreased in formulations containing the two different surfactants, suggesting that the drug contributes to stability through electrostatic repulsion.

Concerning the zeta potentials of NLCs with BTB, it is noteworthy that the zeta values obtained from NLCs formulated with Synperonic^TM^ PE/F68 did not reach zeta values below 30 mV. It is known that surfactants of the Poloxamer 188 type confer protective effects due to the hydrophilic corona present in their structure, resulting in zeta potential values that may not be sufficient for electrostatic stabilization. Through electron microscopy, Bhattacharya and co-authors found that Kolliphor^®^ P188 provides additional steric and electrostatic stabilization of formulations. The presence of this surfactant enhances steric stabilization on the particle surface, even in cases where the charge may be insufficient to generate electrostatic stabilization [31].

According to Shah and co-authors, NCLs can demonstrate enhanced stability, even with a lower zeta potential value, attributed to steric repulsion. This repulsion may result from the adsorption of polymers or non-ionic surfactants on the lipid matrix surface, effectively preventing potential particle aggregation [32].

### 3.5. Nanoparticle Tracking Analysis

NTA is a technique for measuring the size distribution of particles in the sample through the measurement of light scattering properties and Brownian motion. The process involves a laser beam passing through the sampling chamber, where suspended particles in the beam’s path scatter the light. Additionally, it enables the determination of nanoparticle concentration (n° particles/mL) through specific and individual particle counting concerning volume [24].

The results of the NTA analyses are presented in Table 13.

It is observed that all NCLs exhibit particle concentrations in the order of 10^13^ particles/mL, consistent with values found in the literature [15,16,33]. An important parameter to consider in NTA is the Span, which should be lower than one to indicate a homogeneous particle size distribution [24]. Formulations containing Crodasol^TM^ HS HP (CBTB-NLC and C-NLC) displayed a Span value greater than one, indicating a wider range of particle size distribution when compared to Synperonic^TM^ formulations.

When comparing the number of particles between NLCs with BTB and empty NLCs, it is observed that the addition of BTB to the formulations increased the number of nanoparticles in the suspension. The same observation was made by Guilherme and collaborators, who utilized Poloxamer 188 as a surfactant and cetyl palmitate as a solid lipid [15].

### 3.6. Differential Scanning Calorimetry (DSC)

**Figure 9:**
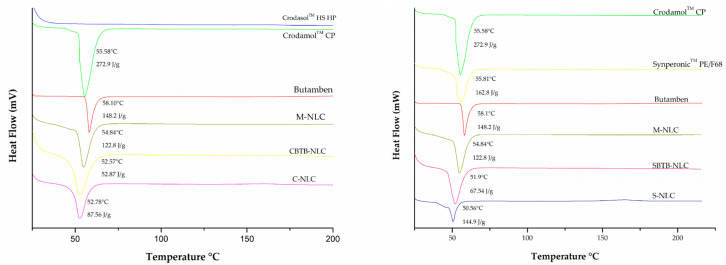
DSC curves of Crodamol^TM^ CP, Crodasol^TM^ HS HP, Synperonic^TM^ PE/F68, butamben, and lyophilized NLCs. CBTB-NLC: NLC with Crodasol^TM^ HS HP and BTB. C-NLC: NLC with Crodasol^TM^ HS HP without BTB. SBTB-NLC: NLC with Synperonic^TM^ PE/F68 and BTB. S-NLC: NLC with Synperonic^TM^ PE/F68 without BTB. M-NLC: a physical mixture of the formulation containing: butamben, Crodamol^TM^ CP, and Synperonic^TM^ PE/F68.

Through this technique, information about the crystallinity of the particles and the interactions between the drug and lipids in the formulation were obtained. Figure 9 displays the DSC curves of the lyophilized NLCs, BTB, Crodamol^TM^ CP, and the two surfactants used. Butamben, Crodamol^TM^ CP, and Synperonic^TM^ PE/F68 exhibited endothermic events at 58.10 °C, 55.58 °C, and 55.81 °C, respectively, corresponding to the melting point of these compounds. These results are in accordance with what is described for these compounds in the literature [22,34,35].

In Figure 9, it can be seen that the C-NLC and CBTB-NLC curves present endothermic events at 52.57 °C and 54.84 °C, respectively. These events are possibly related to the Crodamol™ CP transition in the mixure. When comparing the fusion enthalpy and temperature values, a decrease is noted in both events compared to the pure Crodamol™ CP (55.58 °C, 272.9 J/g). This change can be attributed to the nanometer size of the NLC, as explained by the Gibbs–Thomson effect, which postulates that the smaller the particle size, the greater the surface area, leading to a decrease in the melting point of the NLC compared to the solid lipid due to the presence of surfactants in the NLC. Furthermore, the decrease in the endothermic event of Crodamol™ CP in NLCs may come from an interaction between the drug and Crodamol™ CP (solid lipid), which may alter the lipid’s fusion behavior [36,37,38,39,40].

Similar events are observed in the S-NLC and SBTB-NLC curves (50.56 °C and 51.9 °C). However, in the case of these NLCs, the surfactant used also presents an endothermic event at 55.81 °C (Synperonic™ PE/F68). In these cases, the events observed in NLCs may result from the combination of events related to the solid lipid and the surfactant [40].

When comparing the M-NLC and CBTB-NLC formulations, a decrease in the melting enthalpy and temperature in the CBTB-NLC is observed, which is expected, as the physical mixture is merely all the solid components present in the NLC, mechanically mixed. The same occurs when comparing M-NLC with SBTB-NLC. This indicates that M-NLC presents a more ordered crystal lattice arrangement, unlike NLCs (CBTB-NLC and SBTB-NLC), in which the solid lipid is in less ordered arrangements, contributing to the encapsulation of the drug [37,41,42].

Based on the presented results, it is evident that all NLCs exhibited a decrease in fusion enthalpy, indicating lower lipid crystallinity, minimizing drug expulsion during the storage period [41].

### 3.7. FTIR

Infrared spectroscopy is used to explore the structural characteristics of lipids and identify potential interactions between the drug and the excipients in the formulations [43,44].

**Figure 10:**
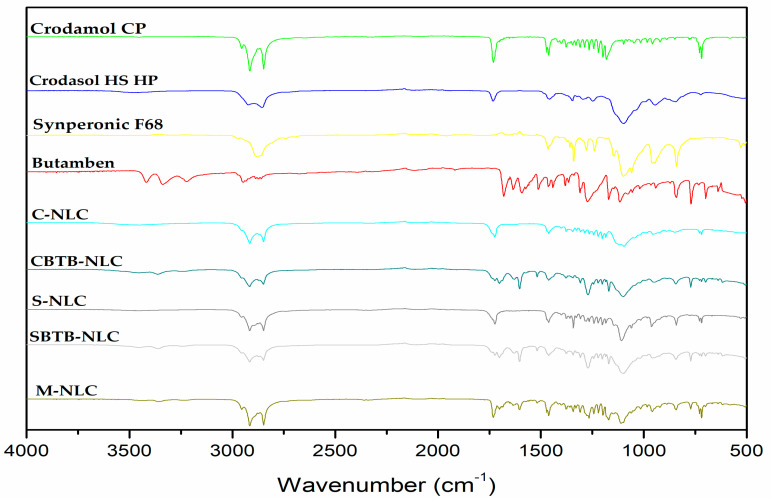
ATR-FTIR absorption spectra of the excipients, lyophilized NLCs, and the drug.

Analyzing Figure 10, it is possible to compare the spectra of the constituents of the formulations with those of the NLCs and with the M-NLC, which is the physical mixture of the developed NLCs. It can be observed that the stretch and deformation signals are consistent with those present in the spectra of Crodamol^TM^ CP, the solid lipid used, and the bands of the surfactants. In the case of S-NLC and SBTB-NLC, their spectra include characteristic bands of their surfactant Synperonic^TM^ PE/F68. The same applies to M-NLC, which has Synperonic^TM^ PE/F68 as a surfactant. Bands in the regions of 1735 cm^−1^, 1250–970 cm^−1^, and 3000–2800 cm^−1^ are present in both Crodamol^TM^ CP and the nanocarrier spectra. Regarding surfactants, bands at 1281–1239 cm^−1^ and 960–942 cm^−1^ are found in the spectra of both surfactants used and also in the spectra of the nanocarriers.

**Figure 11:**
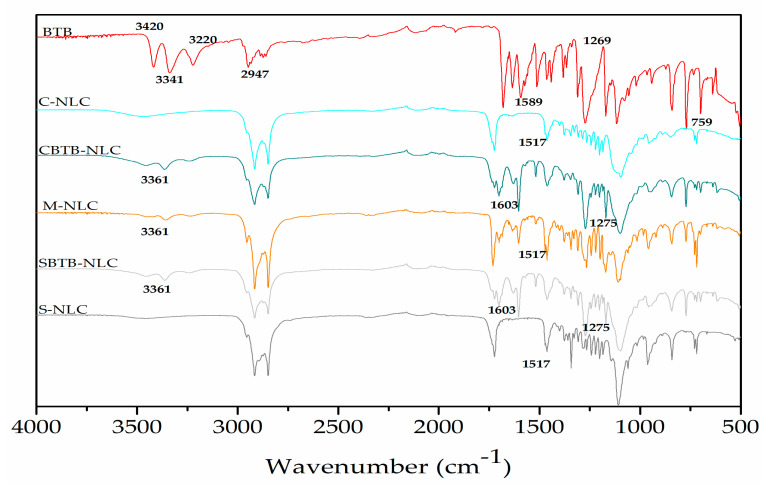
Magnification of the ATR-FTIR absorption spectra of BTB, C-NLC, CBTB-NLC, S-NLC, and SBTB-NLC.

When comparing the BTB spectrum with that of the loaded NLCs (Figure 11), it is evident that the bands at 3420–3341 cm^−1^ (N-H stretching), 1675–1638 cm^−1^ (C=C stretching), and 1589 cm^−1^ (deformation NH2) are absent in the spectra of the loaded NLCs, due to the lower concentration of BTB. However, the bands at 1517 cm^−1^ (C=C stretching—in ring) and 839–700 cm^−1^ (C-H bending and ring puckering deformation) are identifiable in both the drug spectrum and the BTB-NLC spectra.

Analyzing the spectra of BTB-NLCs and M-NLCs (Figure 11), a group of small peaks around 3363–3360 cm^−1^ is observed, which may be a shifted version of the BTB peaks, as it is absent in the spectra of empty NLCs. There is a high similarity between the M-NLC spectra and the CBTB-NLC/SBTB-NLC spectra; however, some small shifts and a decrease in intensity can be seen in the NLC spectra compared to M-NLCs, which may indicate an interaction between the NLC and the drug.

### 3.8. Entrapment Efficiency

Based on the results obtained in this work, the most suitable surfactant was Synperonic^TM^ PE/F6. Therefore, the encapsulation efficiency test was carried out for the optimized formulation using this surfactant. The SBTB-NLC presented 98.45% EE and this result proved that the proposed formulation was an efficient delivery system for this Class II ‘brick-dust’ type of drug. Furthermore, NLCs are capable of encapsulating greater amounts of the drug due to their more disorganized lipid matrix compared to SLNs [39,45].

### 3.9. 3-Month Stability Study of the Optimized NLCs

**Figure 12:**
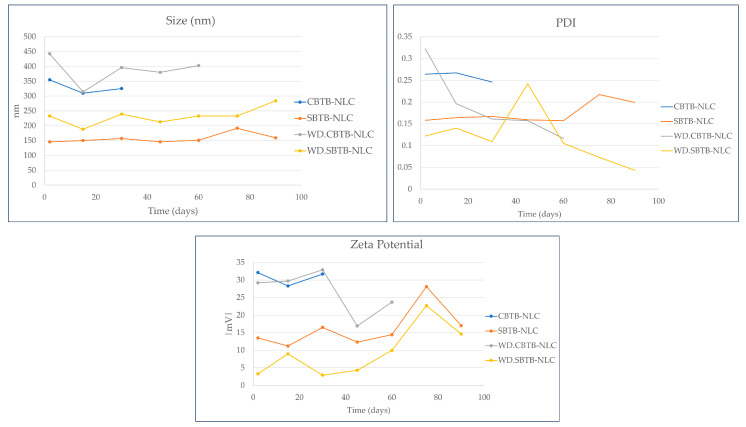
Results of stability analysis of NLCs with butamben.

The evaluated stability parameters were particle size, PDI, and zeta potential. Figure 12 presents the stability results of the formulations.

Based on the results presented in Figure 12, it is observed that in the presence of the drug, the NLCs that have Crodasol^TM^ HS HP in their composition presented shorter stability than NLCs with Synperonic^TM^ PE/F68. This is probably because Synperonic^TM^ PE/F68 provides additional steric and electrostatic stabilization within the NLC and even on the surface of the particles [31].

The study makes us wonder whether it is really important to achieve this type of NLC stability in the form of a dispersion in water since this type of formulation in the liquid state can suffer instability due to oxidation reactions, hydrolysis, phase inversion, crystallization, and polymorphism, which can result in particle aggregation, gelation, and affect drug release [46,47]. Studies report that the ideal approach for an NLC would be to freeze-dry the system or add preservatives, so that long-term stability can be obtained, protecting the NLC from bacterial contamination and maintaining the properties of the nanoparticles, such as particle size, in addition to preventing degradation reactions (hydrolysis) from occurring [28,45,46,47].

## 4. Conclusions

DOE-guided study allowed for optimizing the total lipids, % of surfactant, and sonication time for NLC development, based on the lipid core previously developed [1]. The three surfactants studied, Crodasol^TM^ HS HP, Synperonic^TM^ PE/F68, and Croduret^TM^ 40, presented different behavior regarding the evaluated responses, particle size, PDI, and zeta potential, which was tentatively explained based on their chemical structure.

Comparing Crodasol^TM^ HS HP and Synperonic^TM^ PE/F68, the first resulted in larger particle sizes (244 nm–698 nm), higher PDI values (0.176–0.319), and elevated zeta potential values (|25–29|), in comparison to the second (size: 157–260 nm; PDI: 0.091–0.172; and Zeta: |4.4–19| mV). Croduret^TM^ 40 did not form stable NLCs. Desirability indicated that for Crodasol^TM^ HS HP as a surfactant, the optimized formulation contained 10% total lipids, 5% surfactant, and required 7.5 min of sonication time. For the surfactant Synperonic^TM^ PE/F68, the optimized formulation was defined with 20% total lipids, 10% surfactant, and 10 min of sonication time.

We showed it was possible to incorporate butambem, a ‘brick-dust’ type of drug, within the NLC core and obtain particles with suitable size and polydispersity for Crodasol^TM^ HS HP and Synperonic^TM^ PE/F68, using the optimized percentages and conditions.

Regarding the addition of Super Refined^TM^ DMI to the lipid core, it was observed that this excipient favored the desired NLC properties such as particle size, PDI, and zeta potential, indicating that it remained in the lipid core upon dilution. Moreover, it was of fundamental importance for the solubilization of BTB in the NLC core.

## Figures and Tables

**Table 1:** Experimental variables and design levels.

Variables	Symbols	Low Level (−1)	High Level (+1)
Total lipids (% *w*/*w*)	A	10	20
Surfactant (% *w*/*w*)	B	5	10
Sonication time (min)	C	5	10

**Table 2:** Experimental variables and properties of interest, along with the optimization criteria.

Independent Variables	Dependent Variables (CQAs)	Optimization Criteria for Dependent Variables
% Total lipids	Size	Minimum
% Surfactant	PDI	<0.2
Sonication time (min)	Zeta Potential	Maximize

**Table 3:** Experimental design matrix with the 3 center points.

NLC	A: Total Lipids (%), X_1_	B: Surfactant (%), X_2_	C: Sonication Time (min), X_3_
1	10	5	5
2	20	5	5
3	10	10	5
4	20	10	5
5	10	5	10
6	20	5	10
7	10	10	10
8	20	10	10
9	15	7.5	7.5
10	15	7.5	7.5
11	15	7.5	7.5

**Table 4:** Composition of the formulations and responses obtained in the 2^3^ design for butamben encapsulation, using Crodasol^TM^ HS HP as a surfactant.

NLC	Factor 1	Factor 2	Factor 3	Response 1	Response 2	Response 3
	A: Total Lipids	B: Crodasol^TM^ HS HP	C: Sonication Time	Size	PDI	Zeta Potential
	%	%	min	nm		|mV|
1	10	5	5	244.93	0.187	25.66
2	20	5	5	328.06	0.176	29.33
3	10	10	5	361.76	0.308	26.36
4	20	10	5	698.93	0.319	26.8
5	10	5	10	267.16	0.198	29.16
6	20	5	10	442.7	0.281	27.62
7	10	10	10	364.4	0.317	25.9
8	20	10	10	589.8	0.349	29.26
9	15	7.5	7.5	407.23	0.253	28.16
10	15	7.5	7.5	416.66	0.237	25.4
11	15	7.5	7.5	406.9	0.285	25.76

**Table 5:** Components with significant effects and their interactions on each response for the formulation containing Crodasol^TM^ HS HP as a surfactant.

Response	Positive Effect	Negative Effect
Size	A, B, and AB	BC and ABC
PDI	B	-
Zeta Potential	-	-

**Table 6:** Composition of the formulations and responses obtained in the 2^3^ design for butamben encapsulation, using Synperonic^TM^ PE/F68 as a surfactant.

NLC	Factor 1	Factor 2	Factor 3	Response 1	Response 2	Response 3
	A: Total Lipids	B: Synperonic^TM^ PE/F68	C: Sonication Time	Size	PDI	Zeta Potential
	%	%	min	nm		|mV|
1	10	5	5	260.2	0.139	4.4
2	20	5	5	214.86	0.118	10.73
3	10	10	5	160.9	0.172	8.67
4	20	10	5	157.66	0.134	5.01
5	10	5	10	250.9	0.178	19.73
6	20	5	10	248.4	0.096	14
7	10	10	10	172.06	0.061	6.97
8	20	10	10	162.23	0.11	9.42
9	15	7.5	7.5	171.16	0.109	11.6
10	15	7.5	7.5	188.8	0.145	10.9
11	15	7.5	7.5	194.4	0.131	13.23

**Table 7:** Components with significant effects and their interactions on each response for the formulation containing Synperonic^TM^ PE/F68 as a surfactant.

Response	Positive Effect	Negative Effect
Size	-	B
PDI	-	-
Zeta Potential	C and ABC	B and BC

**Table 8:** Compilation of data from the three experimental designs carried out containing butamben. Orange coloring: positive effect. Blue: negative effect.

Significant Effects			
NLC	Size	PDI	Zeta Potential
Crodasol^TM^ HS HP	% Total lipids, Surfactant, and interactions AB and BC	Surfactant	-
Synperonic^TM^ PE/F68	Surfactant	-	Sonication Time, Surfactant, and interaction BC

**Table 9:** Composition of optimized NLCs.

NLC	CP (g)	Lauryl Lactate (g)	SR^TM^ DMI (g)	Surfactant (g)	BTB (g)	Sonication (min)
CBTB-NLC	0.44	0.28	0.28	0.5	0.5	7.5
C-NLC	0.44	0.28	0.28	0.5	-	7.5
SBTB-NLC	0.88	0.56	0.56	1	0.5	10
S-NLC	0.88	0.56	0.56	1	-	10

Legend. CP—Crodamol^TM^ CP. BTB—butamben. CBTB-NLC: NLC with Crodasol^TM^ HS HP and BTB. C-NLC: NLC with Crodasol^TM^ HS HP without BTB. SBTB-NLC: NLC with Synperonic^TM^ PE/F68 and BTB. S-NLC: NLC with Synperonic^TM^ PE/F68 without BTB.

**Table 10:** Composition of the optimized formulations tested without SR^TM^ DMI.

NLC	CP (g)	Lauryl Lactate (g)	Surfactant (g)	BTB (g)	Sonication (min)
WD.CBTB-NLC	0.61	0.39	0.5	0.5	7.5
WD.SBTB-NLC	1.22	0.78	1	0.5	10

Legend. CP—Crodamol^TM^ CP. BTB—butamben. WD.CBTB-NLC: NLC with Crodasol^TM^ HS HP and BTB and without SR^TM^ DMI. WD.SBTB-NLC: NLC with Synperonic^TM^ PE/F68 and BTB, without SR^TM^ DMI.

**Table 11:** Particle size, PDI, and zeta potential results for the optimized formulations with and without SR DMI^TM^.

NLC	Size (nm)	PDI	Zeta Potential (mV)
CBTB-NLC	354.8	0.264	−32.1
WD.CBTB-NLC	442.8	0.322	−29.2
SBTB-NLC	145.6	0.158	−13.5
WD.SBTB-NLC	233.1	0.122	−3.24

Legend. CP—Crodamol^TM^ CP. BTB—butamben. CBTB-NLC: NLC with Crodasol^TM^ HS HP and BTB. WD.CBTB-NLC: NLC with Crodasol^TM^ HS HP without SR^TM^ DMI. SBTB-NLC: NLC with Synperonic^TM^ PE/F68 and BTB. WD.SBTB-NLC: NLC with Synperonic^TM^ PE/F68 without SR^TM^ DMI.

**Table 12:** Physicochemical characterization of NLCs optimized with and without butamben.

NLC	Size (nm)	PDI	Zeta Potential (mV)
CBTB-NLC	331.3	0.264	−32.1
C-NLC	54.5	0.275	−2.47
SBTB-NLC	166.3	0.16	−9.28
S-NLC	118.3	0.205	−4.43

**Table 13:** NTA analyses results.

NLC	Size (nm)	N° Part. X 10^13^ mL^−1^	D10 (nm)	D50 (nm)	D90 (nm)	Span
CBTB-NLC	175.5 ± 7.2	3.52 ± 0.74	113.2 ± 6.8	137.7 ± 6.6	305.3 ± 50.9	1.39
C-NLC	96.3 ± 24.4	277 ± 0.78	53.5 ± 5.6	74.1 ± 11.3	183.8 ± 67.7	1.75
SBTB-NLC	162.7 ± 2.9	4.40 ± 0.6	130.5 ± 2.1	155 ± 2.1	203.1 ± 3.3	0.46
S-NLC	170.2 ± 1.9	2.82 ± 0.22	135.1 ± 1.2	166.3 ± 3.8	211.5 ± 1.2	0.46

Legend. CP—Crodamol^TM^ CP. BTB—butamben. CBTB-NLC: NLC with Crodasol^TM^ HS HP and BTB. C-NLC: NLC with Crodasol^TM^ HS HP without BTB. SBTB-NLC: NLC with Synperonic^TM^ PE/F68 and BTB. S-NLC: NLC with Synperonic^TM^ PE/F68 without BTB.

## Data Availability

The data used in this study are available to interested by contacting the author M.C.B. (marciacb@unicamp.br).
